# Heart in the crossfire, from epsilon to beyond: cardiac sarcoidosis—a case report

**DOI:** 10.1093/ehjcr/ytaf632

**Published:** 2025-11-29

**Authors:** Humaid Ali, Hamat Hamdi Che Hassan, Mohd Shawal Faizal Mohamad, Boon Cong Beh

**Affiliations:** Faculty of Medicine, Universiti Kebangsaan Malaysia, Jalan Yaacob Latif, Bandar Tun Razak, Cheras, Kuala Lumpur 56000, Malaysia; Department of Medicine, Hospital Canselor Tuanku Muhriz, Universiti Kebangsaan Malaysia, Jalan Yaacob Latif, Bandar Tun Razak, Cheras, Kuala Lumpur 56000, Malaysia; Faculty of Medicine, Universiti Kebangsaan Malaysia, Jalan Yaacob Latif, Bandar Tun Razak, Cheras, Kuala Lumpur 56000, Malaysia; Department of Medicine, Hospital Canselor Tuanku Muhriz, Universiti Kebangsaan Malaysia, Jalan Yaacob Latif, Bandar Tun Razak, Cheras, Kuala Lumpur 56000, Malaysia; Faculty of Medicine, Universiti Kebangsaan Malaysia, Jalan Yaacob Latif, Bandar Tun Razak, Cheras, Kuala Lumpur 56000, Malaysia; Department of Medicine, Hospital Canselor Tuanku Muhriz, Universiti Kebangsaan Malaysia, Jalan Yaacob Latif, Bandar Tun Razak, Cheras, Kuala Lumpur 56000, Malaysia; Faculty of Medicine, Universiti Kebangsaan Malaysia, Jalan Yaacob Latif, Bandar Tun Razak, Cheras, Kuala Lumpur 56000, Malaysia; Department of Medicine, Hospital Canselor Tuanku Muhriz, Universiti Kebangsaan Malaysia, Jalan Yaacob Latif, Bandar Tun Razak, Cheras, Kuala Lumpur 56000, Malaysia

**Keywords:** Cardiac sarcoidosis, Sarcoidosis, Epsilon wave, Ventricular tachycardia, Immunosuppression, Case report

## Abstract

**Background:**

Sarcoidosis is a great mimicker of various medical conditions, which leads to obstacles in early diagnosis and appropriate timely management.

**Case summary:**

A 66-year-old Indian female with metabolic syndrome was initially treated for decompensated liver disease. Her baseline electrocardiogram showed right bundle branch block with a first-degree heart block. She presented 3 months later with angina and heart failure (HF) symptoms, complicated with ventricular tachycardia (VT) treated with i.v. amiodarone and anti-failure medication. Her coronary angiogram revealed mild disease, and her echocardiography showed a mildly reduced ejection fraction (EF) of 45% with regional wall motion abnormalities. Cardiac magnetic resonance imaging (CMR) revealed non-specific left ventricular (LV) patchy mid-wall to epicardial late gadolinium enhancement. Endomyocardial biopsy was complicated with cardiac tamponade and required pericardiocentesis followed by dual-chamber implantable cardioverter-defibrillator (ICD) later. Unfortunately, biopsy result was inconclusive, and serum angiotensin-converting enzyme was within the normal range. She had multiple admissions for the past 2 years for recurrent VT and decompensated HF despite the optimization of ICD setting and guideline-directed medical therapy. Repeated echocardiogram revealed similar EF with thinning of the LV basal septal segment. Her positron emission tomography (PET) scan (Tc-99 m) showed diffuse uptake at the LV myocardium and supraclavicular/mediastinal/abdominopelvic lymph nodes with a mismatch of fluorodeoxyglucose uptake at the basal–inferolateral segment (non-specific). Lymph node biopsy revealed chronic non-caseating granulomatous inflammation. Clinical diagnosis of cardiac sarcoidosis was made based on a histologic diagnosis of extracardiac sarcoidosis with cardiomyopathy/ventricular arrhythmia combined with PET/CMR findings.

**Discussion:**

Cardiac sarcoidosis can have a myriad of symptoms, which may mimic several other disorders leading to a diagnostic challenge.

Learning points

- Cardiac sarcoidosis is a multisystemic condition that can be challenging to diagnose due to the variable presentation
- A high index of suspicion with the use of advanced cardiac imaging techniques including cardiac MRI, PET scan, and perfusion scan can aid in diagnosis and monitoring of treatment response.

## Introduction

Sarcoidosis is a multisystemic chronic granulomatous condition with diverse clinical presentations depending on the organ involved. Cardiac sarcoidosis (CS) itself can present with a myriad of clinical manifestations and electrocardiographic and echocardiographic findings, which can make the diagnosis challenging. We present a case of an elderly woman who initially presented with non-specific constitutional symptoms and subsequently developed unstable arrhythmias. She was eventually diagnosed to have systemic sarcoidosis with cardiac involvement. She required multiple non-invasive and invasive diagnostic investigations prior to definitive diagnosis, resulting in delayed recognition and recurrent hospitalizations.

## Summary figure

**Table:** 

August 2022	Initial presentation with symptomatic ascites
November 2022	Presented with angina and heart failure (HF) symptoms Developed ventricular tachycardia (VT), which was medically managed Subsequently developed bradycardia Cardiac magnetic resonance imaging (MRI) suggestive of infiltrative diseases Endomyocardial biopsy complicated with cardiac tamponade, dual-chamber implantable cardioverter-defibrillator (ICD) inserted
December 2022–August 2024	Endomyocardial biopsy inconclusive Refused for further investigations or procedures Multiple admissions for HF, arrhythmias with ICD shock
August 2024	During another admission for decompensated HF, repeated echocardiography suggestive of CS Agreed for further investigations after counselling Fluorodeoxyglucose (FDG) positron emission tomography–computed tomography (PET–CT) and perfusion scan was suggestive of sarcoidosis
November 2024	Not keen for repeat endomyocardial biopsy, proceeded with lymph node biopsy Histopathological examination suggestive of sarcoidosis
December 2024	Started on prednisolone
May 2025	Repeat FDG PET–CT showed improvement, with improvement of clinical symptoms and admissions

## Case presentation

**Figure 1:**
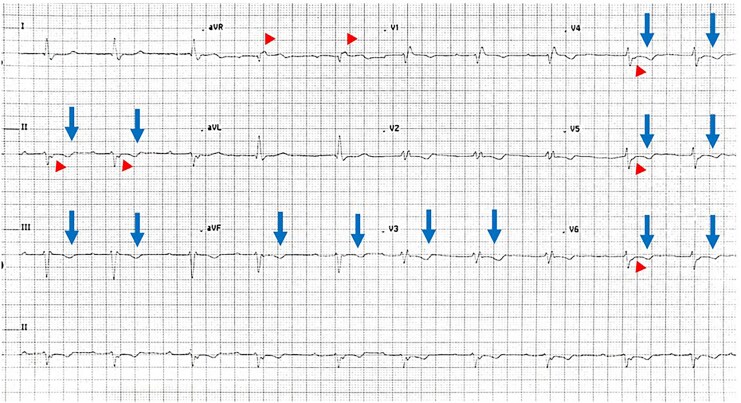
Electrocardiogram showing a small deviation or blip following QRS complex (Epsilon wave)—arrowhead, with non-specific T wave inversions in the inferior and lateral chest leads (arrows).

A 66-year-old Indian female with hypertension, Type 2 diabetes, and dyslipidaemia initially presented with symptomatic ascites and constitutional symptoms including loss of appetite and weight over several months. She reported no angina, palpitations, or symptoms of HF. Investigations at that time suggested metabolic-associated fatty liver disease, and she was discharged with symptomatic treatment. Three months later, she presented with reduced effort tolerance and non-specific chest discomfort. She was admitted and treated for unstable angina and decompensated HF. Electrocardiogram (ECG) on admission showed first-degree atrioventricular block, right bundle branch block, and left anterior fascicular block. There was also an Epsilon wave with non-specific T wave inversions present in the ECG, which was noted (*Figure 1*).

**Figure 2:**
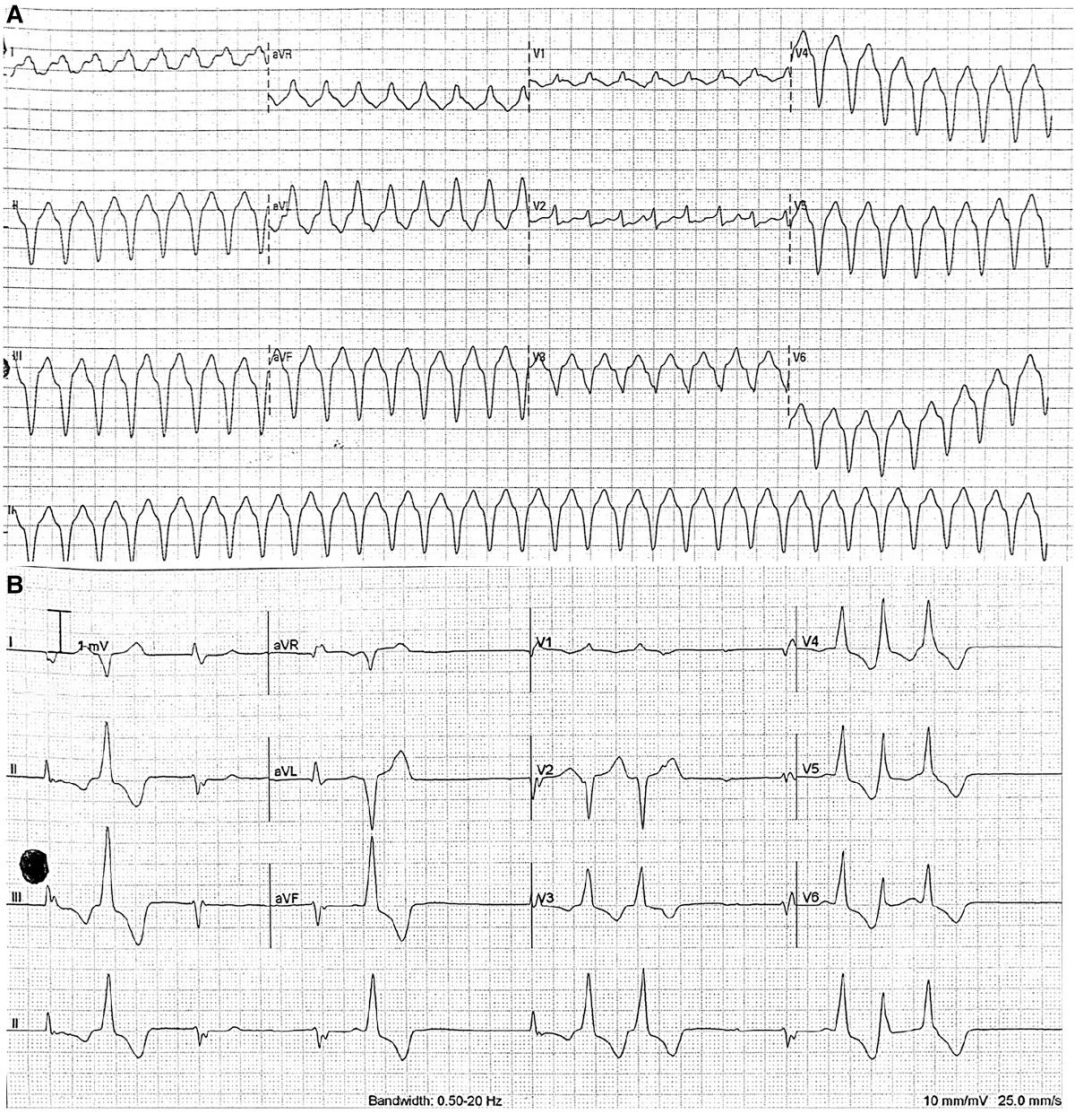
The first electrocardiogram shows monomorphic ventricular tachycardia with possible origin from the inferoseptal region (*A*), with subsequent electrocardiogram post amiodarone (*B*) showing bradycardia with premature ventricular contractions with non-sustained ventricular tachycardia. The morphology of the premature ventricular contractions suggests the origin is from the basal anterolateral wall of the left ventricle. PVCs, premature ventricular contractions.

During admission, she developed palpitations, and ECG showed VT (*Figure 2*). As she was haemodynamically stable, she was treated medically with i.v. amiodarone. Her ECGs during different episodes of VT were suggestive of different origins of VT (*Figure 2A*). Initial echocardiography revealed mildly reduced ejection fraction (EF) of 40%–45% with hypokinetic septal and anterior wall with no obvious valvular abnormalities. After treatment with amiodarone, she had multiple episodes of non-sustained VT with bradycardic episodes (*Figure 2B*). A transvenous temporary pacemaker was inserted in view of bradycardia episodes for overdrive pacing, and a dual-chamber ICD insertion with staged procedure for VT ablation was planned. Coronary angiography was performed at the same time which revealed mild coronary artery disease.

**Figure 3:**
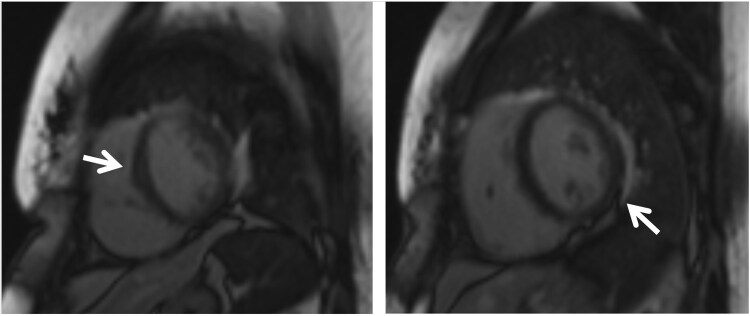
Cardiac magnetic resonance imaging showed patchy mid-wall to epicardial late gadolinium enhancement (arrow) at the apical anterior, basal anteroseptal/inferolateral, anterolateral, inferoseptal, mid-inferoseptal, mid-inferior, and apex, which was suggestive of non-ischaemic causes including infiltrative heart disease such as cardiac sarcoidosis or myocarditis. No CMR evidence to suggest arrhythmogenic right ventricular cardiomyopathy was seen. There was reduced ejection fraction of 26.3% and wall motions of all segments were normal.

During that time, the working diagnosis was arrhythmogenic right ventricular cardiomyopathy (ARVC). A cardiac MRI was performed prior to ICD insertion, which showed reduced EF (35%). However, no MRI features of ARVC were found; instead, patchy late gadolinium enhancement (LGE) was seen, which suggested infiltrative heart disease such as sarcoidosis (*Figure 3*). Her serum calcium and angiotensin-converting enzyme (ACE) levels were normal.

Endomyocardial biopsy and ICD insertion were planned for the same day, but the biopsy was complicated with pericardial tamponade, delaying ICD insertion; pericardiocentesis was performed, and she required prolonged hospital stay for stabilization. Dual-chamber ICD insertion was performed after recovery. Unfortunately, the biopsy result was inconclusive due to limited myocardial tissue in the sample.

**Figure 4:**
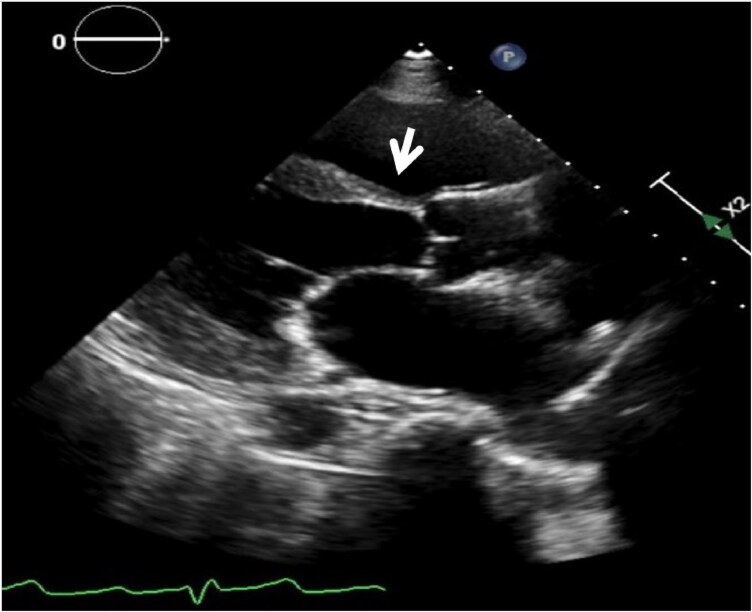
Transthoracic echocardiogram shows thinning of the basal segment of the interventricular septum.

Due to the traumatic experience in the hospital stay, the patient declined further investigation or imaging. Over the next 2 years, she had six admissions with dyspnoea on exertion and peripheral oedema, with crepitation on lung auscultation and elevated jugular venous pressure and was treated as acute decompensated HF. Guideline-directed HF medications including beta-blockers, sodium-glucose co-transporter 2 inhibitors, mineralocorticoid receptor antagonists, and sacubitril/valsartan were titrated as tolerated and optimized. She had recurrent arrhythmias with ICD shocks due to short VT runs and atrial fibrillation while on maintenance amiodarone and apixaban. Repeat echocardiogram revealed reduced EF of 40%–45% with global hypokinesia with minor regional variation with thinning of the basal septal segment (*Figure 4*; Supplementary material online, *Videos*). The valves were normal and the right heart function within normal range and not dilated. Patient and family were re-counselled and agreed for nuclear cardiac imaging for diagnostic evaluation.

**Figure 5:**
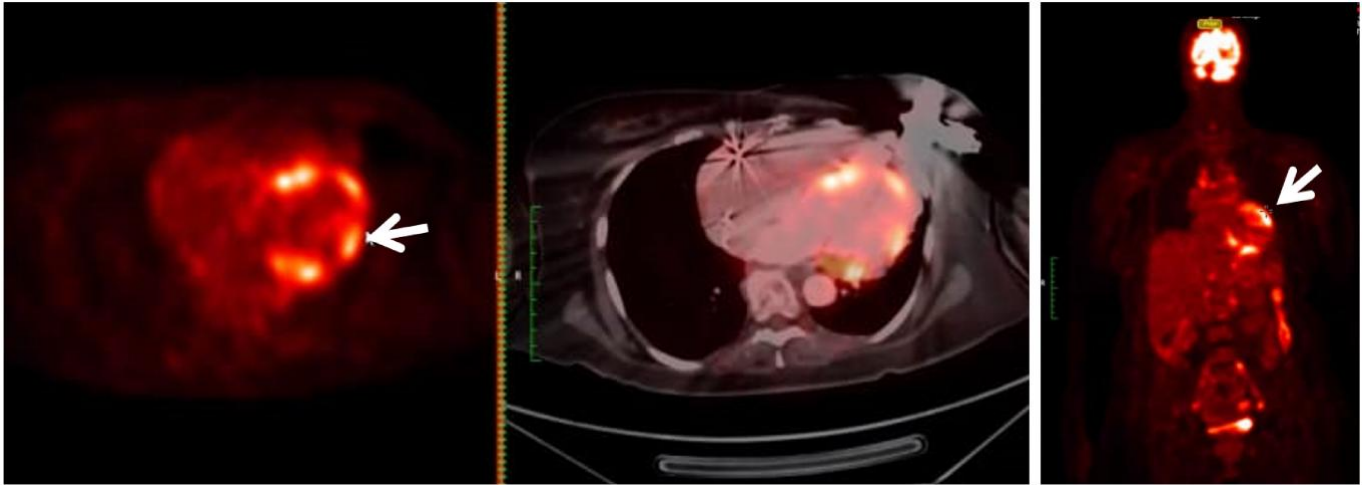
Fluorodeoxyglucose positron emission tomography–computed tomography showed diffuse but non-specific fluorodeoxyglucose uptake on the myocardium (arrow).

Fluorodeoxyglucose positron emission tomography–computed tomography myocardium showed FDG-avid mediastinal, right hilar, bilateral supraclavicular, and abdominopelvic lymphadenopathy with diffuse FDG myocardial uptake (*Figure 5*). Perfusion scan with Tc-99 m sestamibi single photon emission computed tomography–computed tomography (SPECT–CT) showed a non-specific perfusion defect at the basal inferolateral and septal segments, suggesting CS (see Supplementary material online, *Figure S1*). As the patient was not keen for a repeat endomyocardial biopsy, a left supraclavicular lymph node biopsy was performed, which revealed chronic non-caseating granulomatous inflammation (see Supplementary material online, *Figure S2*), confirming the diagnosis of systemic sarcoidosis.

With the histologically confirmed extracardiac sarcoidosis, arrhythmias including VT, echocardiographic findings of basal septal thinning, MRI showing LGE, and PET–CT and perfusion scan findings, the patient fulfilled most international society diagnostic criteria for CS. She was started on oral prednisolone 60 mg once daily (1 mg/kg) with subsequent tapering two weekly. After 6 months, she demonstrated marked improvement in clinical wellbeing, reduced N-terminal pro-B-type natriuretic peptide (NT-proBNP) from 5093 to 639 pg/mL (normal range < 125 pg/mL in sinus rhythm), and fewer ICD shocks and hospitalizations. The follow-up FDG PET–CT showed improvement in previous lesions after 6 months of steroid treatment (see Supplementary material online, *Figure S3*).

## Discussion

Cardiac sarcoidosis is an idiopathic inflammatory cardiomyopathy characterized by non-caseating granulomas of the myocardium. The reported prevalence of CS is 10 per 100 000 in western countries, compared to 1–5 per 100 000 in non-western population,^1^ likely an underestimate due to diagnostic challenges. Cardiac sarcoidosis typically presents as part of multisystemic manifestation of sarcoidosis, which may involve almost any organ system, leading to diverse and often misleading clinical presentations. The most common clinical manifestation of CS includes various degrees of atrioventricular block, VT, HF, and sudden cardiac death.^2^ Electrocardiographic findings such as Epsilon wave, as in our chase, are non-specific, as they may also be seen in other conditions such as ARVC,^3^ making diagnosis challenging.

As the presentation of CS is variable and definitive diagnosis of CS requires endomyocardial biopsy—which is invasive and associated with complications—several societies (Heart Rhythm Society, Japanese Cardiology Society, and World Association of Sarcoidosis and Other Granulomatous Disorders) have proposed diagnostic criteria for CS.^1^ The histological diagnosis of non-caseating granulomatous inflammation on an endomyocardial biopsy remains the gold standard for diagnosis of CS across all three society criteria.^1^ However, as our case demonstrates, the procedure carries significant risks and may have low yield due to patchy involvement of the myocardium. Obtaining extracardiac histological confirmation of sarcoidosis and exclusion of alternative diagnosis is therefore essential for the clinical diagnosis of CS under all three criteria. The additional criteria are based on rhythm abnormalities, echocardiographic findings, and advanced imaging such as cardiac MRI, FDG PET, and perfusion scans.^1^

Advanced imaging including cardiac MRI and FDG PET provides valuable information regarding diagnosis and can be used during follow-up. Cardiac MRI can be used to assess myocardial structure, function, and presence of inflammation and fibrosis. Late gadolinium enhancement is the key finding indicating inflammation and fibrosis and reflects chronicity of myocardial injury.^3,4^ The LGE distribution is usually multifocal and patchy, often involving left ventricular (LV) segments, and the right ventricular side of the septum is usually involved,^3,4^ corresponding to thinning on echocardiography. While CMR indicates chronic inflammation and fibrosis, FDG PET is used to assess active inflammation as inflammatory cells in sarcoid granulomas demonstrate avid glucose uptake.^4^ Increased FGD uptake or ‘hot spot’ with a corresponding perfusion (‘mismatch pattern’), as in our patient, is a characteristic of CS.^3,4^ These perfusion defects are attributed to the scarring or reversible impairment of microcirculation associated with the inflammation in CS.^4^ These findings are also attributed with poor prognosis with increased risk of arrhythmias and sudden cardiac death.^3^ Choosing the appropriate imaging modality is vital as the findings vary depending on the stage of inflammation and fibrosis throughout the course of treatment.

After immunosuppressive treatment is initiated, response should be monitored using a multimodal approach, including clinical symptoms, arrhythmia frequency, hospitalizations, quality of life, and imaging parameters. Fluorodeoxyglucose positron emission tomography has been considered the gold standard for objectively assessing immunosuppressive response in CS.^1^ It has been recommended that FDG PET scan be repeated 6 months after treatment initiation to guide the immunosuppressive adjustment. Serial serum biomarkers including NT-proBNP can provide an objective assessment of HF during follow-up.

## Conclusion

Cardiac sarcoidosis is challenging to diagnose, manage, and monitor due to its variable clinical presentation, reliance on invasive biopsies for histological confirmation, and the cost and complexity of advanced imaging modalities. Management requires a multimodal approach involving anti-arrhythmic therapy, ICD implantation, HF management, and immunosuppressive medications, each carrying potential complications. Early recognition with appropriate imaging is essential to avoid diagnostic delays, enable timely management, and improve outcomes and quality of life.

## Supplementary Material

ytaf632_Supplementary_Data

## Data Availability

The data underlying this article are available in the article and in its online Supplementary material.

## Lead author biography

Dr Humaid Ali is currently completing his internal medicine training at the Hospital Canselor Tuanku Muhriz, Universiti Kebangsaan Malaysia. He is passionate about medical education and expanding knowledge in the novel therapies in the field of medicine.

## Notes

**Consent:** Written consent for submission and publication of this case report including images and associated text has been obtained from the patient, in line with the COPE guidelines.

**Funding:** No financial assistance was provided for writing of this case report.
