# Supplementary figures and images for: Heart in the crossfire, from epsilon to beyond: cardiac sarcoidosis—a case report
Source: Eur Heart J Case Rep. 2025 Nov 29;10(1):ytaf632. doi: 10.1093/ehjcr/ytaf632 (PMC12813286; doi:10.1093/ehjcr/ytaf632)

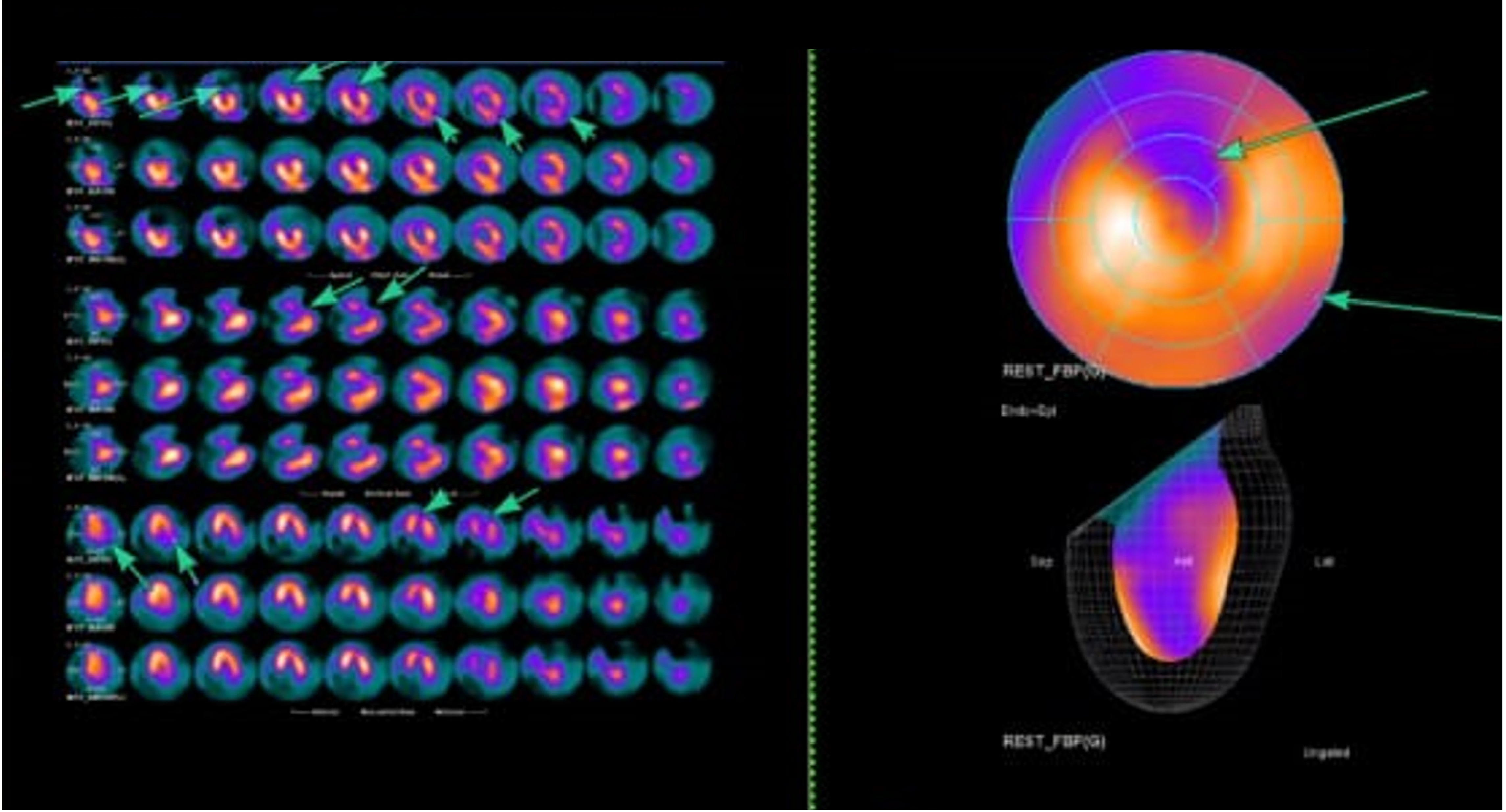

Supplement: ytaf632_Supplementary_Data [file ytaf632_supplementary_data.zip › Supplementary Figure 1.jpg]

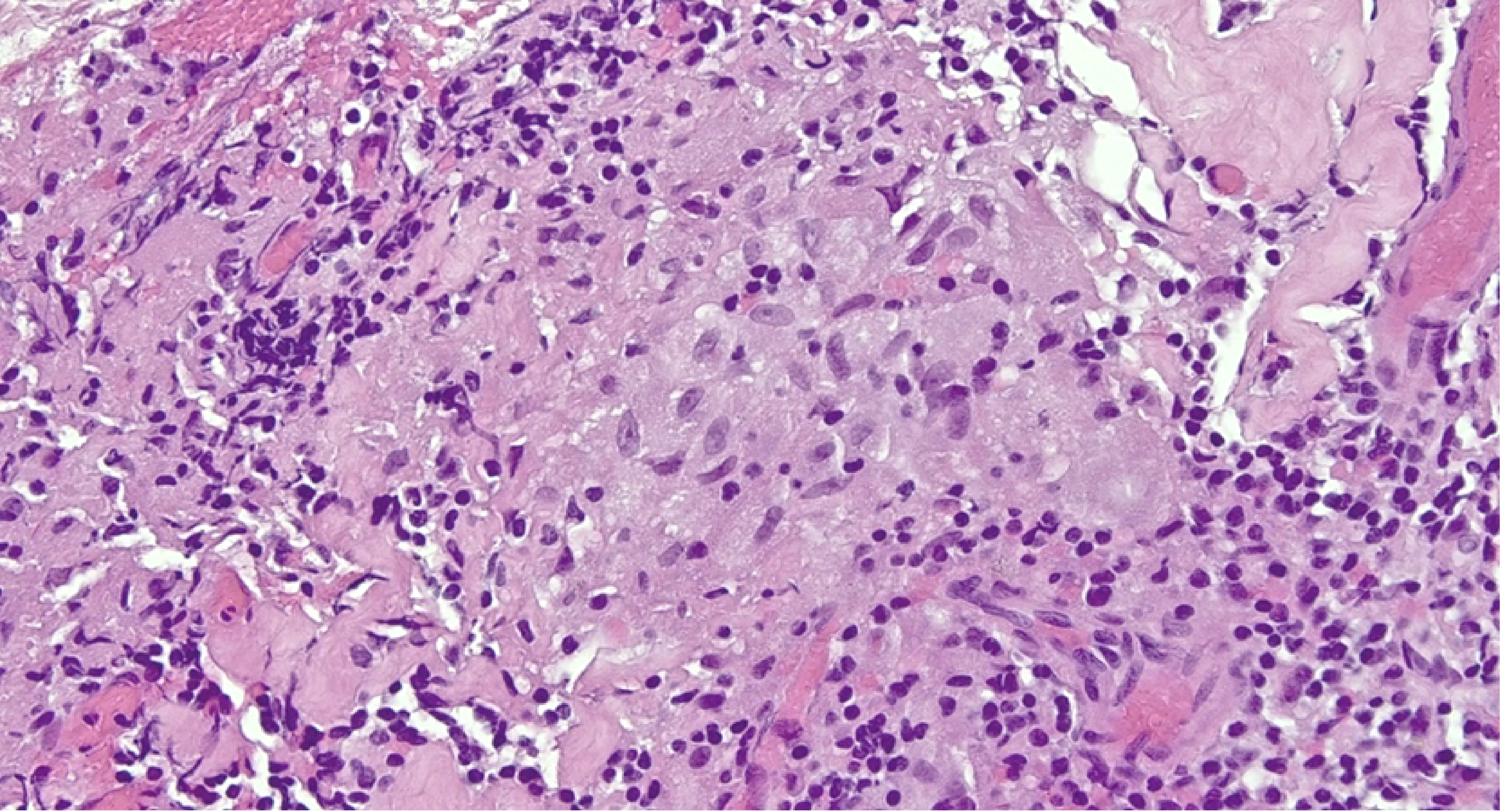

Supplement: ytaf632_Supplementary_Data [file ytaf632_supplementary_data.zip › Supplementary Figure 2.jpg]

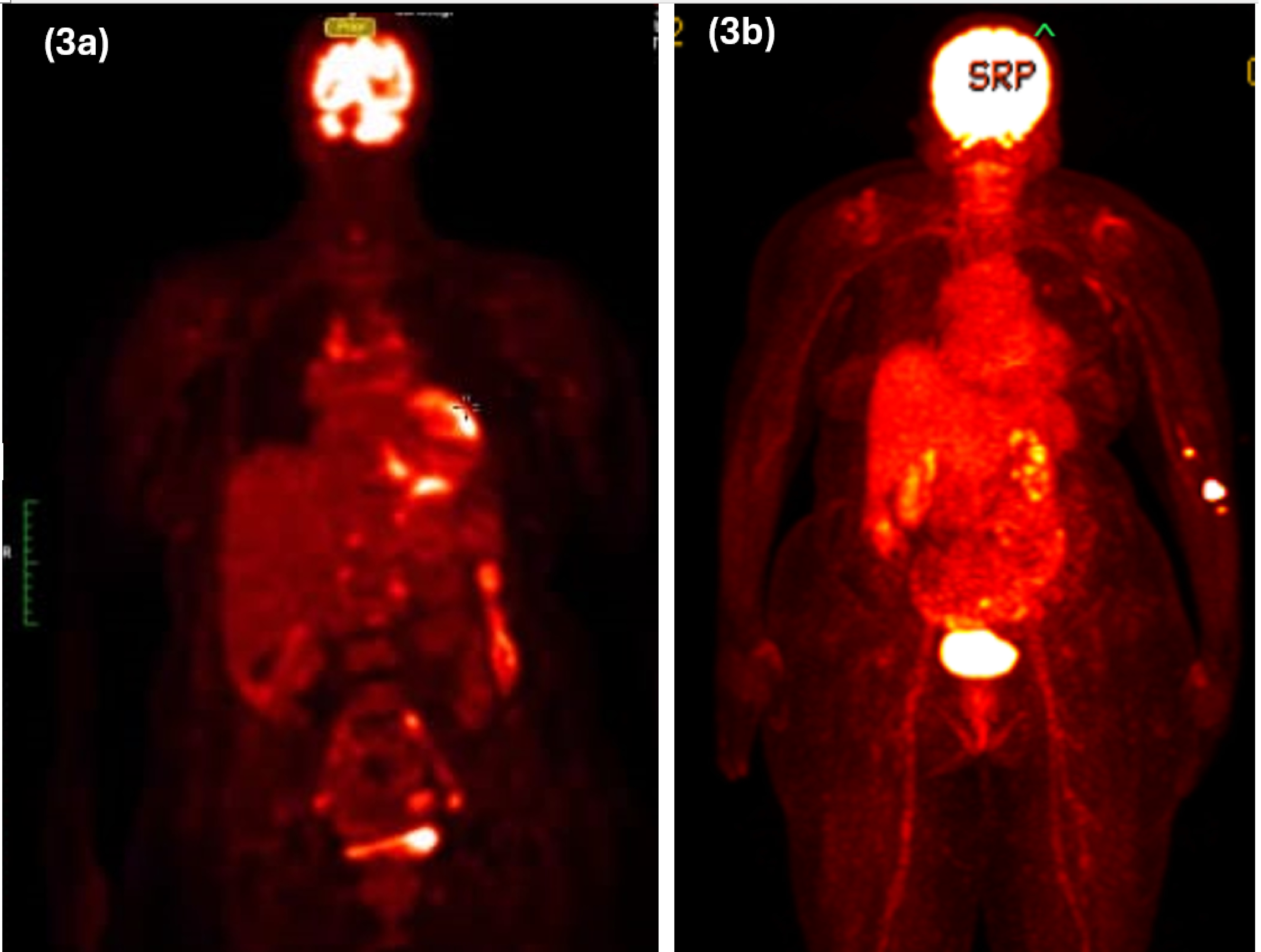

Supplement: ytaf632_Supplementary_Data [file ytaf632_supplementary_data.zip › Supplementary figure 3.jpg]
